# Clinical immunotherapy in pancreatic cancer

**DOI:** 10.1007/s00262-024-03632-6

**Published:** 2024-03-02

**Authors:** Xiaorong Ye, Yue Yu, Xiaohu Zheng, Hongdi Ma

**Affiliations:** 1 Department of Gastroenterology, The First Affiliated Hospital of USTC, Division of Life Sciences and Medicine, University of Science and Technology of China, Hefei, 230001 Anhui Province People’s Republic of China; 2 Department of Neurosurgery, The First Affiliated Hospital of USTC, Division of Life Sciences and Medicine, University of Science and Technology of China, Hefei, 230001 Anhui Province People’s Republic of China; 3 Hefei National Research Center for Physical Sciences at Microscale, The CAS Key Laboratory of Innate Immunity and Chronic Disease, School of Basic Medical Sciences, Center for Advanced Interdisciplinary Science and Biomedicine of IHM, Division of Life Sciences and Medicine, University of Science and Technology of China, Hefei, People’s Republic of China; 4 Key Laboratory of Quantitative Synthetic Biology, Shenzhen Institute of Synthetic Biology, Shenzhen Institute of Advanced Technology, Chinese Academy of Sciences, Shenzhen, People’s Republic of China; 5 Department of Pediatrics, The First Affiliated Hospital of USTC, Division of Life Sciences and Medicine, University of Science and Technology of China, Hefei, 230001 Anhui Province People’s Republic of China

**Keywords:** Pancreatic cancer, Chemotherapy, Immunotherapy, Monotherapy, Combination therapy

## Abstract

Pancreatic cancer remains a challenging disease with limited treatment options, resulting in high mortality rates. The predominant approach to managing pancreatic cancer patients continues to be systemic cytotoxic chemotherapy. Despite substantial advancements in immunotherapy strategies for various cancers, their clinical utility in pancreatic cancer has proven less effective and durable. Whether administered as monotherapy, employing immune checkpoint inhibitors, tumor vaccines, chimeric antigen receptors T cells, or in combination with conventional chemoradiotherapy, the clinical outcomes remain underwhelming. Extensive preclinical experiments and clinical trials in the realm of pancreatic cancer have provided valuable insights into the complexities of immunotherapy. Chief among the hurdles are the immunosuppressive tumor microenvironment, limited immunogenicity, and the inherent heterogeneity of pancreatic cancer. In this comprehensive review, we provide an overview and critical analysis of current clinical immunotherapy strategies for pancreatic cancer, emphasizing their endeavors to overcome immunotherapy resistance. Particular focus is placed on strategies aimed at reshaping the immunosuppressive microenvironment and enhancing T cell-mediated tumor cell killing. Ultimately, through deeper elucidation of the underlying pathogenic mechanisms of pancreatic cancer and the refinement of therapeutic approaches, we anticipate breakthroughs that will pave the way for more effective treatments in this challenging disease.

## Introduction

Pancreatic cancer, ranking as the seventh leading cause of cancer-related fatalities, remains a formidable malignancy characterized by a grim prognosis and a mortality-to-incidence ratio of 94% [1]. Although there has been a modest improvement in its 5-year survival rate in recent years, a lack of breakthrough treatment options persists. Several factors contribute to the dismal outlook for pancreatic cancer patients. Firstly, the absence of early diagnostic markers or discernible clinical symptoms suitable for pancreatic tumor screening often results in the diagnosis of locally advanced or metastatic disease at the time of presentation. Secondly, treatment options for pancreatic cancer are exceedingly limited, further compounding the poor prognosis. Conventional treatments, such as FOLFIRINOX (comprising fluorouracil, leucovorin, irinotecan, and oxaliplatin) or a combination of nab-paclitaxel plus gemcitabine, afford a median overall survival (OS) of merely 11.1 and 8.5 months, respectively, for patients in good health who receive these standard systemic chemotherapy regimens [2–4]. Surgery represents the sole potential cure for this disease; however, a mere 20% of patients diagnosed with localized pancreatic cancer are eligible candidates for surgical intervention. Furthermore, recurrence of pancreatic cancer remains commonplace, even among patients who receive standard adjuvant therapy following surgery [5]. Consequently, there exists an urgent imperative for novel therapeutic strategies in the battle against pancreatic cancer.

Immunotherapy, in essence, entails the augmentation of the human immune system's capacity to recognize and eliminate tumor cells through various mechanisms. Its overarching objective is the complete eradication of malignant cells while sparing normal cell function. Immunotherapy has spawned numerous drug regimens grounded in diverse mechanisms and has exhibited remarkable success in the realm of malignancies such as metastatic melanoma and hematological tumors. Nevertheless, its clinical utility in solid tumors, notably pancreatic cancer, has been less auspicious, though this does not preclude its promising potential [6].

In this comprehensive review, we provide an overview of current clinical immunotherapies for pancreatic cancer, elucidate their underlying mechanisms, and assess their efficacy. Furthermore, we spotlight technological advancements, encompassing improvements in antibody technology, the manipulation and amplification of cancer-killing cells, and cancer vaccines, all of which have injected innovation into the field of immunotherapy and hold the promise of a brighter future in the battle against pancreatic cancer.

## Immune checkpoint-based treatment options

**Fig. 1:**
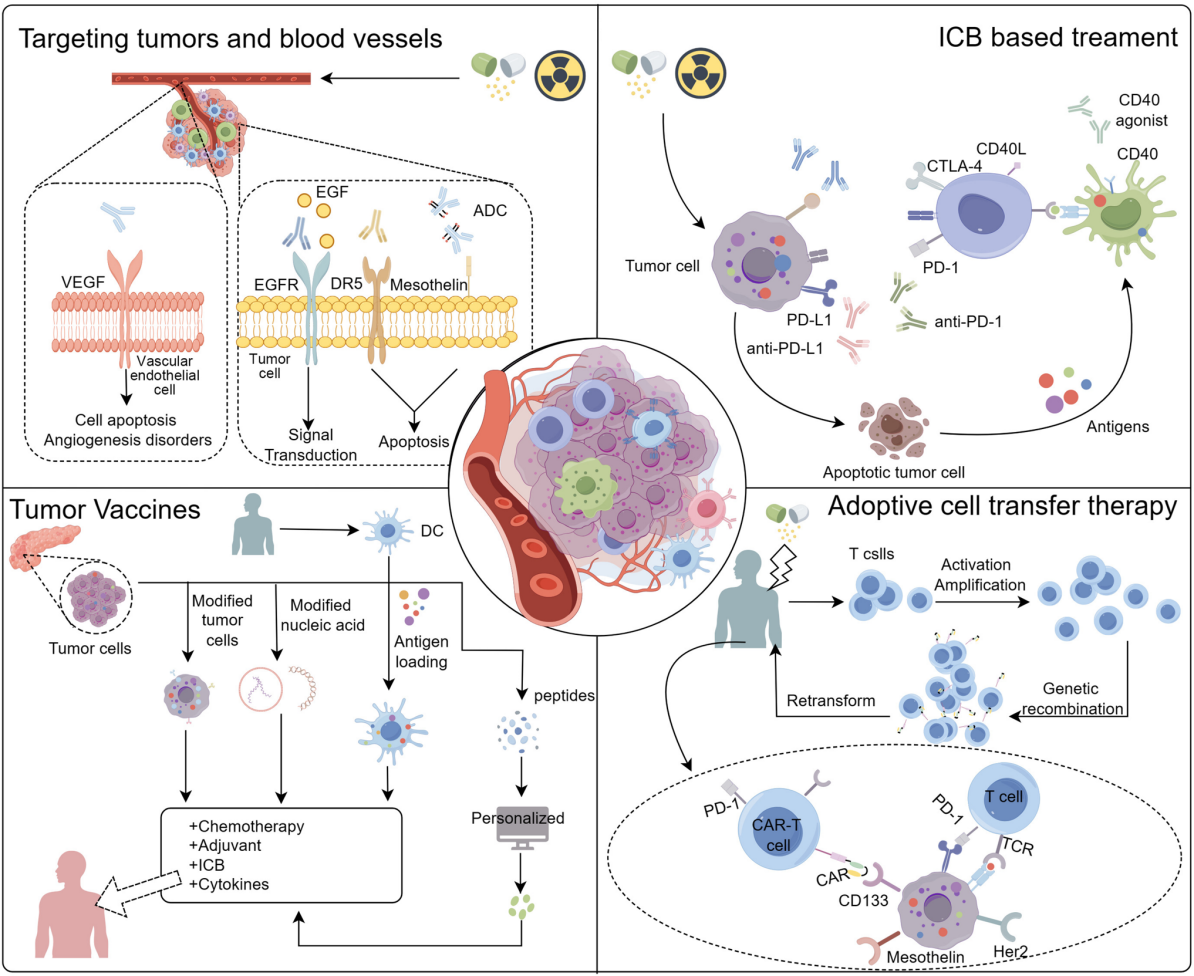
Current clinical immunotherapy strategies for pancreatic cancer. This schematic shows the monotherapies and combined therapies for pancreatic cancer. The clinical immunotherapy strategies for pancreatic cancer are shown as monotherapy, including administrated with tumors or angiogenesis-targeted antibodies, immune checkpoint inhibitors, tumor vaccines, chimeric antigen receptor (CAR) T cells, and also as in combination with chemotherapy or radiotherapy. *Upper left*: Monoclonal antibodies that target tumors or angiogenesis, inhibit tumor development, and promote apoptosis of tumor cells. They are usually used in combination with chemotherapy or radiotherapy. *Upper right:* Treatment options based on ICB. These treatments are usually used in combination with drugs that inhibit tumor growth, improve the tumor microenvironment, and also in combination with conventional chemotherapy or radiotherapy. *Bottom left:* Whole cell vaccines, peptide vaccines, dendritic cell vaccines, and nucleic acid vaccines are prepared differently. Tumor vaccines are often used in combination with chemotherapy, ICB, etc. *Bottom right:* T cells are extracted from the patient's body and genetically modified to express chimeric antigen receptors (CARs), enabling them to mount a more potent and sustained response. These engineered T cells circulate within the body, delivering precise and efficient anti-tumor capabilities

T cells represent indispensable components of the human immune system, regulated by costimulatory and inhibitory signals as they engage in the recognition and elimination of tumor cells in vivo [7, 8]. Immune checkpoints constitute a category of immunosuppressive molecules that operate under physiological conditions to maintain self-tolerance and prevent the body from inadvertently harming its healthy cells during infection or excessive inflammation [9–11]. Research has revealed that tumor cells can elude immune cell recognition and destruction by binding to immune checkpoints on the surface of immune cells, thereby effecting immune evasion [12–15]. Therapeutic strategies centered on disrupting the interaction between tumor cells and immune cell checkpoints hold the potential to reverse immune evasion. Notably, PD-1 (Programmed cell death protein 1) and PD-L1 (Programmed death-ligand 1), as well as CTLA-4 (cytotoxic T-lymphocyte-associated protein 4), were the first immune checkpoints to be discovered, garnering significant attention as therapeutic targets [15] **(**Fig. 1**)**. Corresponding monoclonal antibodies can prevent PD-1 from binding with PD-L1 and PD-L2, as well as inhibit the binding of PD-L1 to CD80 protein, thereby reinstating the immune system's ability, particularly that of T cells, to target and eliminate tumor cells, while preserving normal immune function [16, 17]. Similarly, by disrupting the ligand-receptor interactions of B7-1/B7-2 and CTLA-4, these interventions enable effector cells to sustain their recognition and killing functions.

**Table 1:**
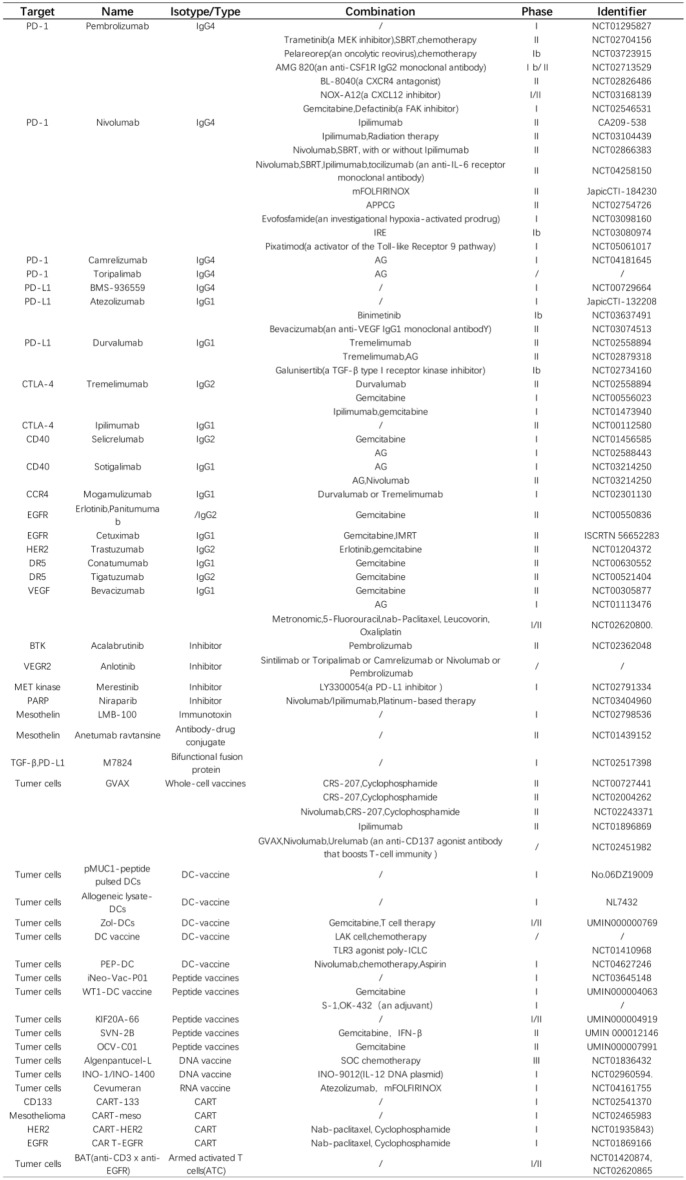
Clinical trials for immunotherapy-related regimens in pancreatic cancer

In addition to immune checkpoint inhibitors, another class of drugs has garnered attention due to its distinct mechanism of action. CD40, a member of the TNF (tumor necrosis factor) receptor superfamily, exhibits wide distribution among antigen-presenting cells and plays a pivotal role in activating antigen-presenting cells and enhancing T cell-mediated anti-tumor immunity. Consequently, CD40 represents a compelling antagonistic target [18–20] **(**Fig. 1**)**. Activation of CD40 sets in motion tumor suppression through various pathways in diverse CD40 agonist-based combination therapy regimens. For instance, CD40-activated macrophages enhance the intratumoral concentration of chemotherapeutic drugs by disrupting the extracellular matrix. Moreover, CD40 agonists heighten the efficacy of the adaptive immune system against tumor cells by augmenting antigen presentation, bolstering CD8 + T cell activity, and fostering immune cell infiltration at tumor sites, effectively transforming "cold" tumors into immunologically active environments [19].

In summary, the discovery of immune checkpoints and the evolution of clinical applications involving immune checkpoint inhibitors and agonists have broadened the landscape of anti-tumor treatment options, charting novel directions for anti-tumor research and bring about a resurgence in the exploration of anti-tumor immunity (Clinical trials of drugs targeting immune checkpoints are listed in Table 1).

### Monotherapy using single immune checkpoint drugs or combinations

Blocking the PD-1 and PD-L1 axis or preventing CTLA-4 binding to its ligands with monoclonal antibodies has demonstrated definitive efficacy in many patients with solid tumors [21–24]. However, the clinical effectiveness of this monotherapy approaching pancreatic cancer patients has been disappointing. Currently, the following immune checkpoint drugs are commonly employed in clinical trials and therapies for pancreatic cancer: PD-1 inhibitors such as pembrolizumab, nivolumab, camrelizumab, and toripalimab; PD-L1 inhibitors like atezolizumab and durvalumab; and CTLA-4 inhibitors including ipilimumab and tremelimumab.

Pembrolizumab (MK-3475), a potent PD-1-specific IgG4-κ humanized monoclonal antibody, obstructs the binding of PD-1 to its ligands, PD-L1 and PD-L2. In a phase I study, pembrolizumab exhibited variable efficacy among different advanced solid tumor patient populations, with both pancreatic cancer patients experiencing disease progression during treatment [25].

Similarly, clinical trials of anti-PD-L1 monotherapy have yielded discouraging results. An early phase I trial involving seven pancreatic cancer patients treated with BMS-936559, a high-affinity, IgG4 human-derived anti-PD-L1 monoclonal antibody, failed to elicit any partial responses (PR) [26]. In another phase I study, one pancreatic cancer patient achieved progression-free survival (PFS) for 12.2 months following treatment with atezolizumab, an engineered immunoglobulin monoclonal antibody targeting PD-L1, showing promise but lacking sufficient evidence to draw definitive conclusions [27].

Conversely, clinical trials of anti-CTLA-4 monotherapy have produced even more disheartening outcomes, with no evidence of efficacy and the emergence of unmanageable side effects. In a phase II study of 27 patients with locally advanced or metastatic pancreatic cancer treated with ipilimumab, a fully humanized CTLA-4 antibody, only 40% (eight of 20) completed the treatment course due to disease progression and side effects. Notably, three immunological adverse events of grade ≥ 3 were observed, one of which resulted in the patient's death. According to the response evaluation criteria in solid tumors, it was concluded that single-agent ipilimumab, at a dose of 3.0 mg/kg, was an effective therapy for pancreas adenocarcinoma [28].

Furthermore, combination therapy involving anti-PD-1/L1 and anti-CTLA-4 demonstrated limited efficacy in pancreatic ductal adenocarcinoma (PDAC), in contrast to its success in other solid tumors [29, 30]. O'Reilly EM et al. conducted a phase II randomized clinical trial in patients with metastatic PDAC, administering a combination of durvalumab, a human anti-PD-L1 monoclonal antibody, and tremelimumab, a human anti-CTLA-4 monoclonal antibody. The objective response rate (ORR) was 3.1% for patients receiving combination therapy and 0% for those receiving monotherapy [31].

Interestingly, the combination therapy of anti-PD-L1 and anti-CTLA-4 demonstrated some efficacy in pancreatic neuroendocrine tumors (pNETs). In a study involving a combination of ipilimumab and nivolumab, an anti-PD-1 monoclonal antibody, three out of seven (43%) pNET patients achieved objective responses in CA209-538, particularly those with high-grade tumors, including one previously refractory to single-agent anti-PD-1 therapy [32]. This suggests that patients with high-grade pNETs can significantly benefit from dual checkpoint blockade.

In summary, immune checkpoint inhibitor monotherapy, with limited data, has demonstrated little to no impact on overall prognosis in pancreatic cancer patients. However, the clinical benefit observed in individual patients suggests the therapeutic potential of this approach, particularly in vaccinated patients who exhibit stronger responses to immune checkpoint drugs [28]. Additionally, the combination strategies of anti-CTLA-4 plus anti-PD-1 or anti-PD-L1 have shown anti-tumor activity, especially in pNETs, although further clinical trials are required to establish their potential value. These findings underscore the need for combination therapy strategies based on immune checkpoint drugs to combat pancreatic cancer effectively.

### Immune checkpoint drugs combined with chemotherapy

#### anti-PD-1 combined with (m) FOLFIRINOX or gemcitabine-based chemotherapy

Both the combination of anti-PD-1 with (modified) FOLFIRINOX and gemcitabine-based chemotherapy have demonstrated superior efficacy compared to chemotherapy alone.

A phase II study involving a combination of modified FOLFIRINOX, a standard adjuvant therapy for patients with resectable and advanced disease [3, 33–36], and nivolumab as a first-line treatment for 31 metastatic pancreatic cancer patients yielded promising results. FOLFIRINOX was modified by having a lower dose of fluorouracil compared to standard FOLFIRINOX. This regimen resulted in a median OS of 13.40 months (90% CI 10.87–15.24) and a median PFS of 7.39 months (90% CI 3.88–7.59), with a 1-year survival rate of 54.8% (90% CI 39.1–68.1%). Notably, the most frequently reported grade 3–4 drug-related adverse event was neutrophil count decrease (38.7%) [37]. Overall, the addition of a PD-1 antibody to (m) FOLFIRINOX has shown slight improvements over the known efficacy of (m) FOLFIRINOX alone [38, 39].

Furthermore, the CISPD-4 study, a randomized phase II trial investigating the combination of PD-1 antibodies with modified FOLFIRINOX for borderline resectable and locally advanced pancreatic cancer, is currently underway [40], which may provide additional positive data for this combination therapy regimen.

The combination therapy of anti-PD-1 with gemcitabine and nab-paclitaxel (AG) chemotherapy as a first-line treatment has also shown promising efficacy. In a previous study, this combination achieved a remarkable 100% disease control rate (DCR) in patients with advanced pancreatic cancer as a first-line therapy [41]. Patients in this study achieved a median OS of 15 months and a median PFS of 9.1 months. Similar encouraging data regarding DCR were observed in other clinical trials. In a single-arm, single-center exploratory study that enrolled 20 metastatic PDAC patients, camrelizumab, an anti-PD-1 antibody, combined with AG as a first-line therapy resulted in a 60% ORR and an 85% DCR [42]. Similarly, in a phase II trial, 80% of patients with advanced pancreatic cancer achieved an ORR when treated with NAPPCG (N/nivolumab plus AP/albumin-bound paclitaxel plus P/paricalcitol and plus C/cisplatin + G/gemcitabine) as a first-line therapy, with a median PFS of 8.2 months [43].

The trial data mentioned above suggest that the combination therapy of anti-PD-1 with gemcitabine-based chemotherapy elicits a stronger initial response in pancreatic cancer patients. However, investigating methods to sustain this anti-tumor response and prolong patients' survival remains a key area for further research. Notably, a case report documented a patient with metastatic PDAC who achieved durable responses and tolerated well a triple combination therapy of toripalimab (a humanized IgG4K monoclonal antibody specific for human PD-1), gemcitabine, and nab-paclitaxel, even though pseudoprogression was observed in this patient, suggesting that some patients may derive substantial benefit from this protocol [44].

#### anti-CTLA-4 combined with gemcitabine

The safety of tremelimumab, a human monoclonal IgG2 antibody targeting CTLA-4, in combination with gemcitabine has been established in patients with metastatic pancreatic cancer [45]. However, compared to PD-1, the results of the combination of CTLA-4 and chemotherapy have been less optimistic. In a phase I study conducted in 2020, patients with advanced pancreatic cancer treated with ipilimumab plus gemcitabine achieved a median PFS of 2.5 months (95% CI 0.8–4.8) and a median OS of 8.5 months (95% CI 2.2–10.3), which did not surpass the efficacy of gemcitabine monotherapy. Common grade 3 or 4 adverse events included anemia (48%), leukopenia (48%), and neutropenia (43%) [46].

The combination of anti-CTLA-4 and chemotherapy does not appear to demonstrate superiority over the combination of PD-1 with chemotherapy, which may be attributed to differences in chemotherapy regimens and the varying therapeutic effects of anti-CTLA-4 and anti-PD-1 [47].

#### anti-PD-1 plus anti-CTLA4 plus chemotherapy

In contrast to the combination of anti-CTLA-4 with gemcitabine, the addition of PD-1 and CTLA-4 antibodies to chemotherapy has shown better survival benefits, albeit with increased side effects. The combination of gemcitabine and nab-paclitaxel (Nab-P) serves as the standard first-line treatment for advanced PDAC. In the recent Canadian Cancer Trials Group PA.7 trial (NCT02879318), a randomized phase II study, the combination of gemcitabine plus nab-paclitaxel with durvalumab and tremelimumab improved patients' OS (9.8 months versus 8.8 months; HR 0.94; P: 0.72) and ORR (30.3% versus 23.0%; OR 1.49; P 0.28) compared to the control group. However, there was no significant difference in PFS (5.5 months versus 5.4 months), and the overall incidence of adverse events in the experimental group was higher (68.91% versus 44.3%) [48].

Based on the results of clinical trials involving the combination of anti-PD-1 or anti-CTLA-4 with chemotherapy, it appears that the three-drug regimen combining anti-PD-1 and anti-CTLA-4 with chemotherapy may not warrant further research for the treatment of pancreatic cancer. This is due to the fact that, in addition to the limited improvement in clinical benefits compared to the two-drug combination regimen, the tolerability of self-toxicity and side effects in advanced pancreatic cancer patients remains a significant concern.

#### CD40 agonist combined with gemcitabine-based chemotherapy

As early as 2011, the combination of CD40 agonistic monoclonal antibodies (mAbs), such as selicrelumab, with gemcitabine was observed to induce tumor regression in advanced pancreatic cancer patients. Among 21 patients, four developed a partial response, 11 had stable disease (SD), and four experienced disease progression (PD). The median PFS for these patients was 5.6 months (95% confidence interval, 4.0 months to not estimable), and the median OS was 7.4 months (95% CI, 5.5 to 12.8 months). This combination demonstrated better clinical benefit than historical gemcitabine monotherapy, highlighting the potential of CD40 agonistic mAbs when combined with chemotherapy [20, 49].

More recently, the safety and tolerability of combination therapy with CD40 agonists and AG (gemcitabine and nab-paclitaxel) have been demonstrated. In an open-label, multicenter, phase Ib study involving APX005M (sotigalimab), another CD40 agonistic monoclonal antibody, combined with chemotherapy, 14 out of 24 patients with metastatic pancreatic adenocarcinoma responded positively, with eight of them receiving additional nivolumab. Common grade 3–4 treatment-related adverse events included decreased lymphocyte counts (20; 67%) and decreased neutrophil counts (9; 30%). The most common serious adverse event was pyrexia (6 [20%] of 30) [50]. Overall, based on the observed tolerability and clinical activity, this study suggests a viable combination therapy option for advanced pancreatic cancer patients.

However, in the recently published phase II results of this study, the experimental group receiving AG plus sotigalimab with or without nivolumab did not exhibit improved efficiency compared to the control group. The 1-year OS rates for the two groups were 48.1% (sotigalimab plus chemo, *p* = 0.062, *n* = 36) and 41.3% (sotigalimab, nivo plus chemo, *p* = 0.223, *n* = 35), respectively. There was no significant improvement observed in ORR or PFS in either arm [51, 52]. This lack of improvement may be attributed to functional exhaustion following T cell hyperactivation and potential efficacy antagonism introduced by the combination therapy. While the results of this study are negative, they provide crucial insights for future research into combination therapy strategies and the underlying immune mechanisms of pancreatic cancer.

Furthermore, the addition of CD40 agonists to neoadjuvant chemotherapy has demonstrated immunological changes and clinical benefits. In one study, selicrelumab, an agonistic CD40 mAb, was added to the standard chemotherapy regimen of gemcitabine and nab-paclitaxel before surgery for 16 patients with resectable pancreatic cancer. This combination proved to have tolerable toxicity, resulting in an OS of 23.4 months (95% CI, 18.0–28.8 months). Additionally, immunological changes, such as increased proliferation and activation of T cells in the tumor microenvironment (TME) and a decrease in M2-like tumor-associated macrophages, were observed in postoperative patients compared to preoperative untreated patients [53]. Focusing on these immunological changes within the TME under these treatments may pave the way for future studies aimed at overcoming the current efficacy challenges faced by regimens combining CD40 agonists with chemotherapeutics.

For patients with pancreatic cancer, the combined treatment strategy of immune checkpoint drugs with chemotherapy offers certain advantages over chemotherapy alone. However, the contradiction between chemotherapy drugs' immune system-suppressing effects and the immune system's dependence on immune checkpoint drugs may limit the efficacy of this combination strategy. Additionally, the rapid progression of pancreatic cancer and the immune cell exhaustion in its late stages weakens the immune system's anti-tumor capabilities, thereby limiting the clinical impact of immune checkpoint drugs. Furthermore, the potential autoimmune toxicity resulting from ICIs or CD40 agonists, as well as the tolerability of combination therapy, is important considerations before initiating treatment, particularly for patients with abnormal autoimmune function or those who have undergone multiple rounds of chemotherapy.

### Combination of immune checkpoint drugs with other drugs

In pancreatic cancer, the immune microenvironment characterized by immunosuppression presents a significant challenge to the efficacy of immunotherapy. Simultaneously, targeting different nodes involved in anti-tumor immunosuppression holds the theoretical promise of producing a synergistic effect.

#### Anti-PD-1 combined with anti-colony-stimulating factor-1

Colony-stimulating factor-1 (CSF-1) is produced by various tumors and recruits cells like myeloid-derived suppressor cells (MDSCs) to promote immunosuppression. Preclinical data in a pancreatic cancer mouse model suggest that blocking CSF-1/CSF-1R signaling can lead to anti-tumor T cell responses and tumor regression [54, 55]. However, the effectiveness of anti-PD-1 combined with anti-CSF1R therapy in pancreatic cancer remains inconclusive. A study involving solid tumor patients, including those with pancreatic cancer, treated with 200 mg pembrolizumab plus 1100 mg AMG 820, an anti-CSF1R monoclonal antibody, did not yield sufficiently robust anti-tumor activity to warrant further investigation [56].

#### Anti-PD-1/L1 combined with transforming growth factor β receptor inhibitor

Transforming growth factor β (TGF-β) is a multifunctional cytokine in the transforming growth factor superfamily that promotes immunosuppression, angiogenesis, tumor cell epithelial-to-mesenchymal transition (EMT), and other tumor-promoting processes. Blocking the TGF-β pathway may sensitize tumors to immune checkpoint inhibitors by affecting immune cell function [57–59]. Preclinical studies have shown that TGF-β receptor inhibitors (TGFβ-RI) can efficiently block the TGF-β canonical pathway [60–62], and dual inhibition of TGFβ-RI and PD-L1 may synergistically enhance anti-tumor efficacy by downregulating PD-1 expression while relieving TGF-β's inhibitory effects on CD8 + T cells [63, 64]. However, a two-part, single-arm, multinational, phase Ib study combining galunisertib, a TGF-β type I receptor kinase inhibitor, with durvalumab in recurrent/refractory metastatic pancreatic cancer patients reported inconsistent results. The study showed a median OS of 5.72 months (95% CI: 4.01 to 8.38) and PFS of 1.87 months (95% CI: 1.58 to 3.09), with a DCR of 25.0% and a confirmed ORR of 3.1% [65].

Similarly, clinical trials targeting both PD-L1 and TGF-β pathways have yielded pessimistic results. M7824 (MSB0011359C), a novel bifunctional fusion protein consisting of a human IgG1 monoclonal antibody against PD-L1 fused to the extracellular domain of TGF-β receptor II (TGF-βRII), was tested in a phase I study with patients suffering from solid tumors. While durable and confirmed partial responses were observed in one of five patients (20%) with pancreatic cancer [66], the overall results were not promising.

#### Anti-PD-L1 combined with vascular endothelial growth factor (VEGF) inhibitor

VEGF and its receptors are essential regulators of angiogenesis under normal physiological conditions. VEGF is expressed in various tumors, promoting tumor angiogenesis and metastasis. In the case of pancreatic cancer, most tumors are VEGF-positive, making it a potential target [67]. The combination of anti-PD-L1 with a VEGF inhibitor has shown effectiveness in pNET patients. Halperin DM et al. treated 20 pNET patients with atezolizumab in combination with the VEGF inhibitor bevacizumab. The study reported a PFS of 14.9 months (95% CI, 4.4–32.0) and an ORR of 0%; (95% CI, 5.7%-43.7%) [68].

#### Anti-PD-L1/CTLA-4 combined with anti-CC chemokine receptor 4 mAb

CC chemokine receptor 4 (CCR4), expressed in various tumor and Treg cells, has been shown to increase the number of CD56 + NK cells and induce potent ADCC (antibody-dependent cellular cytotoxicity)-mediated anti-tumor effects as well as decrease the number of Foxp3 + Treg cells in peripheral in a tumor-bearing mouse model when targeted by its monoclonal antibody [69–72]. However, clinical trials investigating the combination of anti-CCR4 and anti-PD-L1 at tolerated levels have not shown significant efficacy. In a phase I clinical trial, Zamarin D et al. enrolled 64 patients with solid tumors, including 27 with pancreatic cancer, who were treated with mogamulizumab, a humanized, defucosylated immunoglobulin G1 kappa mAb, in combination with durvalumab or tremelimumab. Despite tolerability, the ORR was only 5.3% (95% CI, 0.1%–26.0%), with one patient achieving a partial response [73]. The lackluster results may be attributed to inadequate drug concentration within the local tumor to trigger pharmacological effects or the absence of specific immune cell types in the pancreatic cancer tumor microenvironment, limiting the anti-tumor efficacy of CCR4 mAb. Additionally, the associated depletion of effector T cell populations may counteract the positive therapeutic effects of Treg depletion.

#### Anti-PD-1 combined with inhibitor of CXCR4-CXCL12 axis

The COMBAT trial has shown promising signs of efficacy in combining anti-PD-1 therapy with a CXCR4 inhibitor and chemotherapy for pancreatic cancer patients. This trial enrolled patients with metastatic PDAC who had progressed after first-line therapy. They were subsequently treated with BL-8040 (a CXCR4 antagonist) and pembrolizumab as second-line regimens. The results indicated a median OS of 7.5 months. In the expansion cohort, where the NAPOLI-1 regimen (nanosome irinotecan, fluorouracil, and leucovorin) was added, the ORR, DCR, and median persistence were 32%, 77%, and 7.8 months, respectively, for the experimental group consisting of 22 patients [74]. However, subsequent data for 43 patients treated with this regimen showed a decrease in ORR (21%), DCR (63.02%), and median duration of clinical benefit (5.7 months) compared to previous data. In the intention-to-treat population, median PFS was 3.8 months, and median OS was 6.6 months. Importantly, the triple combination was safe and well-tolerated, with a low incidence of grade 3 or higher neutropenia and infection (7%) [75]. These alterations in efficacy may be attributed to the modulation of tumor immunosuppression in pancreatic cancer.

Conversely, a clinical study involving anti-CXCL12 (NOX-A12) showed positive results. In patients with microsatellite-stable disease, unresponsive to anti-PD-1 therapy, cotreatment with a 2-week start-up period of NOX-A12 followed by pembrolizumab resulted in disease stabilization observed in 2 (22%) of nine patients. Clinical activity was also observed in other patients, characterized by significantly longer treatment duration than before [76]. Given that chemotherapeutic drugs can increase the exposure of tumor neoantigens to the immune environment through direct cell destruction and that inhibition of the CXCR4-CXCL12 pathway can reprogram the tumor microenvironment [77–79], the combination of chemotherapy and CXCR4-CXCL12 pathway blockade holds significant promise.

#### Immune checkpoint drugs combined with kinase inhibitor

Bruton's tyrosine kinase (BTK), a non-receptor kinase, has been implicated in the immunosuppressive function of myeloid-derived cells in the tumor microenvironment [80, 81]. While BTK inhibitors have shown the ability to inhibit pancreatic cancer progression in mouse models [82], the clinical combination therapy of a PD-1 inhibitor with a BTK inhibitor has demonstrated poor efficacy. A randomized phase II study evaluating pembrolizumab and acalabrutinib in comparison with acalabrutinib alone in patients with advanced pancreatic cancer failed to reveal a significant improvement in the ORR (7.9%, 95% CI: 1.7% to 21.4% in the combination therapy arm, versus 0%, 95% CI: 0% to 10%, in the monotherapy arm) and median PFS (1.4 months, 95% CI: 1.3 to 1.4 months, and 1.4 months, 95% CI: 1.3 to 1.5 months) [83]. Furthermore, the study did not assess changes in MDSCs and T cell subsets in the tumor microenvironment.

Similarly, anlotinib, a tyrosine kinase inhibitor targeting VEGF receptors 2, has shown the ability to reduce vascular density in tumor tissue and inhibit tumor growth [84, 85]. However, combining a tyrosine kinase inhibitor (TKI) with PD-1 therapy in pancreatic cancer has not improved patient survival. In an observational, prospective study involving patients with advanced pancreatic cancer, the combination regimen of anlotinib plus anti-PD-1 antibodies resulted in the worst PFS compared to other solid tumors, with a PFS of 1.61 months versus 8.37 months (95% CI: 6.5–10.0 months) [86].

Likewise, the phase Ia/Ib PACT study demonstrated the stable safety and durable clinical activity of LY3300054, a new PD-L1 inhibitor with a modified Fc domain that prevents PD-L1–expressing T cell depletion. However, combinations of this drug with tyrosine-protein kinase (MET) inhibitors have been less effective in patients with pancreatic cancer. After treatment with LY3300054 and merestinib (a type II MET kinase inhibitor), most pancreatic cancer patients experienced disease progression [87]. Moreover, in a recent phase Ib clinical trial, the combination of avelumab (a PD-L1 blocking human IgG1 monoclonal antibody) and binimetinib (a small-molecule MEK1/2 inhibitor) also showed limited clinical activity in metastatic pancreatic ductal adenocarcinoma (mPDAC) patients [88].

#### Immune checkpoint drugs combined with kinase inhibitor and chemotherapy

The incorporation of chemotherapy agents alongside ICIs and kinase inhibitors appears to ameliorate the unfavorable outcome associated with the two-drug combination regimen. Focal adhesion kinase (FAK), a tyrosine kinase overactivated in the majority of PDAC and linked to a poor prognosis, was targeted in a recent multicenter, open-label, phase 1 study. The three-drug combination of defactinib, a small-molecule FAK inhibitor, pembrolizumab, and gemcitabine, demonstrated good tolerability and safety in patients with advanced refractory pancreatic cancer. Notably, among the 10 evaluable patients, one achieved a PR, seven showed SD, and two exhibited PD [89].

#### Immune checkpoint drugs combined with PARP inhibitors and platinum-based therapy

Niraparib, a poly (ADP ribose) polymerase (PARP) inhibitor inhibiting DNA repair and inducing tumor cell death, significantly enhanced progression-free survival in patients with advanced ovarian cancer. In a recent randomized phase Ib/II study of niraparib combined with nabuliumab or ipilimumab in patients with platinum-sensitive advanced pancreatic cancer [90], despite 50% of patients experiencing grade 3–4 treatment-related AEs, the nira/ipi group was deemed superior due to a 59.6% progression-free survival rate at 6 months (PFS6) and a 17.3-month mOS as opposed to the nira/nivo group [91]. This precision targeting strategy may offer personalized immune drug selection for patients with distinct characteristics.

#### Immune checkpoint drugs combined with activator of the Toll-like receptor 9 pathway

Pixatimod, a compound augmenting innate immunity by activating the Toll-like receptor 9 pathway, has been reported to potentially enhance the efficacy of ICIs. Unfortunately, a phase Ib open-label multicenter study of solid tumors treated with nivolumab revealed no responders among 18 mPDAC patients [92]. This implies that augmenting the anti-tumor capability of innate immunity may not eliminate T cell rejection and restriction in advanced PDAC; yet, we believe that enhancing the innate immune system may exert a more potent effect on early-stage pancreatic cancer treatment.

It is apparent that drugs targeting immune system suppression have clinical efficacy limitations. Contributing factors may include:

- Recruitment of patients with a history of multiple failed treatments in trials, potentially leading to unknown changes in tumor-localized microenvironments.
- Heterogeneity in local tumor cellular composition and an insufficient number of recruited patients in studies.
- Persistence of immunosuppressive components in the tumor microenvironment and the emergence of adaptive resistance mechanisms.
- Challenges in achieving adequate drug penetration into local tumor sites and maintaining sustained effects within the tumor microenvironment.

Nevertheless, research in this area warrants attention and ongoing exploration to enhance our comprehension of the local environment and molecular mechanisms of pancreatic cancer.

### Therapeutic regimen based on immune checkpoint drugs and radiotherapy

Radiation therapy is believed to induce a distal anti-tumor immune response through the abscopal effect, enhancing tumor cell sensitivity to immune cell killing. The combination with ICIs may potentially reverse the cold tumor characteristics of pancreatic cancer. In a single-arm, non-randomized, phase 2 trial combining radiation, ipilimumab, and nivolumab in patients with metastatic microsatellite-stable (MSS) PDAC, a response was observed in patients receiving radiation therapy, with a DCR of 29% (5/17; 95%CI: 10–56%), and an ORR of 18% (3/17; 95%CI: 4–43%). This study confirms the ability of radiation therapy to improve the response rate to immunotherapy [93].

The safety of ICIs combined with stereotactic body radiotherapy (SBRT) was demonstrated in a phase I clinical trial [94]. In another randomized phase II study of nivolumab with or without ipilimumab combined with SBRT, there were also positive results for refractory metastatic pancreatic cancer. Compared to patients treated with SBRT/nivolumab, those in the SBRT nivolumab/ipilimumab group achieved a higher clinical benefit rate (CBR) (37.2% vs. 17.1%) and a higher percentage of patients who achieved a PR (14.0% vs. 2.4%). In conclusion, the regimen exhibited clinically significant anti-tumor activity and a good safety profile [95].

Similarly, the addition of anti-PD-1 therapy plus KRAS inhibitor to chemoradiotherapy regimens has demonstrated both safety and improved efficacy when compared to control groups. In a phase II study involving 198 patients with postoperative local recurrence of pancreatic cancer characterized by mutant KRAS and positive immunohistochemical staining of PD-L1, eligible participants received chemotherapy (mFOLFIRINOX or 5-fluorouracil). Following this, they were administered SBRT (35–40 Gy in five fractions), intravenous pembrolizumab (200 mg/3 weeks), and trametinib (2 mg/day) or SBRT (with the same regimen) and intravenous gemcitabine (1000 mg/m(2)) on day 1 and 8 of a 21-day cycle for eight cycles. The results indicated that the group receiving SBRT plus pembrolizumab and trametinib achieved a median OS of 24.9 months (95% CI 23.3–26.5), whereas the control group (SBRT + gemcitabine) had a median OS of 22.4 months (95% CI 21.2–23 6). This demonstrated a notable improvement with a hazard ratio (HR) of 0.60 (95% CI 0.44–0.82; p = 0.0012). The most common grade 3 or 4 adverse effects observed were increased alanine aminotransferase or aspartate aminotransferase (ten [12%] of 85 in SBRT plus pembrolizumab and trametinib group vs. six [7%] of 85 in SBRT plus gemcitabine group) [96].

However, the outcomes of a phase II study examining the effectiveness of a multi-drug combination involving ICIs and radiotherapy for refractory pancreatic cancer were not as promising as anticipated. At the data cutoff, no response was observed in 26 patients treated with ipilimumab, nivolumab, tocilizumab (an anti-IL-6 receptor monoclonal antibody), and SBRT. Five patients (19%; 95%CI, 7–39) demonstrated stability. The median overall survival was 5.3 months (95%CI 2.3–8.0). Furthermore, 19 patients (73%) experienced treatment-related adverse events. This outcome may be associated with the intricate role of IL-6 in the specific tumor microenvironment of pancreatic cancer [97].

In general, the efficacy of ICIs in combination with radiotherapy or chemoradiotherapy met expectations and indeed enhanced the adaptive anti-tumor immune response. However, the effectiveness of incorporating other types of drugs into this combination strategy varies. The amalgamation of precise radiotherapy with more personalized and diversified immunotherapy regimens may represent one of the avenues to unlock the therapeutic potential of ICIs in the future.

### Immune checkpoint drugs plus other treatments

Oncolytic viruses, capable of entering tumor cells and causing persistent killing without harming normal tissues, hold promise for selective anti-tumor effects and promoting anti-tumor immune responses, particularly when utilizing wild-type or gene-edited viruses. Pelareorep, an intravenously delivered oncolytic reovirus, has shown safety and efficacy in combination with chemotherapy for various malignant tumors [98, 99]. However, its application in pancreatic cancer patients has yet to yield positive clinical results. In a phase Ib study, pelareorep and pembrolizumab were added to chemotherapy. This combination led to encouraging efficacy, with three out of 10 patients achieving disease control, one patient experiencing a partial response, and two patients achieving stable disease, which persisted for 9 and 4 months, respectively [100].

In a noncontrolled, single-arm, open-label, phase 2 clinical trial, seven patients with first-line treatment-resistant mPDAC were given oncolytic parvovirus (H-1PV, ParvOryx). Two out of seven achieved PR and lived up to 326 and 555 days, respectively. In addition, ParvOryx was well-tolerated by the patients [101].

Evofosfamide, an investigational hypoxia-activated prodrug, holds promise in the treatment of pancreatic cancer. In a recent phase I dose-escalation study combining ipilimumab with evofosfamide in advanced pancreatic cancer patients, while no patients achieved a confirmed partial response, five out of seven patients exhibited stable disease [102]. The safety of this combination was demonstrated, and responders displayed increased proliferation of peripheral T cells and greater infiltration of intratumoral T cells into the hypoxic tumor microenvironment, providing a basis for further investigation of this combination therapy.

Moreover, immune checkpoint drugs combined with topical therapy, specifically electroporation, have been explored in the context of pancreatic cancer. Depletion of tumor stroma has the potential to enhance the killing of tumor cells by cytotoxic T lymphocytes, thus synergizing with immunotherapy [103, 104]. Irreversible electroporation (IRE), a surgical therapy that directly damages cancer tissue, has also been employed in patients with PDAC [105, 106]. The combination of IRE and anti-PD-1 therapy has demonstrated impressive safety and efficacy. In a phase Ib clinical trial involving 10 patients with stage 4, unresectable pancreatic cancer, two patients did not receive planned treatment and relapsed at 3 months. The remaining eight patients achieved a median PFS of 6.8 months (95% CI 3.5–10.0). The median OS reached 18 months (95% CI 9.2–26.8), with only one patient experiencing a nivolumab-related adverse event. The most common adverse reactions included pain, fatigue, diarrhea, nausea, and hypertension. Encouraged by these positive results, a phase II trial is currently underway [107].

While the combination of ICIs with oncolytic viruses, adjuvants, or electroporation has demonstrated varying clinical effects, it suggests that non-traditional treatment methods may enhance anti-tumor effects within the context of immunotherapy. Research into these effective non-traditional treatments represents a critical aspect of the battle against pancreatic cancer, and further clinical trials are essential to fully realize their potential.

In summary, mounting evidence suggests that monotherapy with anti-PD-1/L1, anti-CTLA-4, and CD40 agonists has limited clinical efficacy in pancreatic cancer patients. Combination strategies involving immune checkpoint blockades and therapies or drugs targeting various mechanisms, particularly radiotherapy and oncolytic viruses, have shown some improvements in outcomes, although the variability and magnitude of improvement remain limited. This may be attributed to the challenging-to-reverse tumor microenvironment of pancreatic cancer, which hinders the sustained action of drugs and anti-tumor immune cells. As a result, efforts are focused on identifying ways to modify the tumor microenvironment of pancreatic cancer, thereby enhancing anti-tumor activity. This includes investigating new mechanisms for inhibiting tumor development, discovering novel immune checkpoints to optimize immune cell anti-tumor functions, and developing additional combination treatment strategies.

## Treatment options targeting tumors and blood vessels

Since the pioneering use of hybridoma technology to create highly specific monoclonal antibodies in 1975 [108], the monoclonal antibody industry has continued to expand and plays an indispensable role in immunology and tumor therapy. Despite their high specificity and pharmacological advantages, monoclonal antibodies face several challenges in their application, such as off-target effects, limitations related to antibody size, and the risk of immune responses. To address these issues, various approaches have been explored, including humanization technology, Fc region modification, the design of drug delivery platforms targeting tumor cells, and the development of bispecific antibodies, all of which show promise [109].

Tumor-associated antigens, typically overexpressed on the surface of tumor cells and under-expressed or absent in normal cells, play crucial roles in tumor development, migration, and intercellular communication [110, 111]. Targeting these antigens can directly inhibit or kill tumors through various mechanisms. The following sections describe four types of monoclonal antibodies targeting tumors and blood vessels currently under investigation in pancreatic cancer clinical research (Fig. 1). Relevant drug information and clinical trials are also listed in Table 1.

### Blocking cell growth factor signaling

#### Epidermal growth factor receptor (EGFR) inhibitor plus gemcitabine

The combination of erlotinib (an oral EGFR inhibitor) and panitumumab (a fully human monoclonal antibody inhibiting EGFR-expressing tumors) with gemcitabine in patients with advanced pancreatic cancer demonstrated improved median OS (8.3 months versus 4.2 months; HR, 0.817; 95% CI, 0.530–1.260; p = 0.1792) and PFS (3.6 months versus 2.0 months; HR, 0.843; 95% CI, 0.555–1.280; p = 0.4190). However, this improved efficacy was accompanied by increased toxicity, with patients in the experimental group experiencing a higher frequency of grade 3 and higher non-hematologic toxicities (82.6% vs. 52.2%; p = 0.0018) [112]. These findings do not support this regimen as a superior treatment option.

#### EGFR inhibitor plus gemcitabine plus radiotherapy

In a prospective phase II study, patients with locally advanced pancreatic cancer (LAPC) received maintenance therapy with gemcitabine or gemcitabine plus cetuximab, a monoclonal antibody targeting EGFR, following a combination therapy consisting of cetuximab, gemcitabine, and intensity-modulated radiation therapy (IMRT). The results indicated that the addition of cetuximab did not improve the survival benefit of patients after chemoradiotherapy, with a 13-month median OS showing no clear advantage compared to historical data [113].

#### EGFR plus HER2 inhibitor plus gemcitabine

In a phase II multicenter study, first-line therapy in advanced pancreatic cancer patients involved gemcitabine, trastuzumab (a monoclonal antibody targeting the HER2 receptor), and erlotinib. The study observed partial tumor responses in 19% of patients, disease stabilization in 56%, and a DCR of 74.6% (95% CI: 61.8–85.0; 44/59 patients). The median PFS was 3.5 months (95% CI: 2.4–3.8), and the median OS was 7.9 months (95% CI: 5.1–10.2) [114]. However, this combination did not demonstrate clear superiority over standard therapy.

### Inducing apoptosis

#### DR5 agonist plus gemcitabine

Death receptor 5 (DR5), a cell surface receptor of the TNF-receptor superfamily, mediates apoptosis. Conatumumab, which binds to and activates DR5, can induce tumor cell apoptosis. In a randomized, placebo-controlled phase II study in patients with metastatic pancreatic cancer, the combination of conatumumab and gemcitabine resulted in a 6-month survival rate of 59% (42–73) compared to 50% (33–64) in the gemcitabine arm, with neutropenia being the most common grade ≥ 3 adverse event [115]. Additionally, a combination of tigatuzumab (a humanized monoclonal antibody activating DR5) and gemcitabine demonstrated clinical activity in a phase II trial involving patients with advanced unresectable pancreatic cancer. The study reported a PFS of 52.5% (95% CI, 39.3–64.1%) at 4 months, with the most common adverse events being nausea (35.5%) and fatigue (32.3%) [116]. Both studies support further research into drugs with apoptosis-inducing mechanism.

### Anti-mesothelin

Mesothelin, an antigen differentially expressed on the surface of tumors and normal cells, is a potentially effective target for pancreatic cancer. LMB-100, a conjugate of a mesothelin antibody and pseudomonas toxin A, demonstrated limited effectiveness in a phase I study involving patients with advanced solid tumors [117]. Notably, its effectiveness in pancreatic cancer patients was suboptimal.

Another multicenter study involving anetumab ravtansine, another antibody conjugate targeting mesothelin, enrolled 148 patients in phase II with various types of solid tumors. None of the patients with pancreatic cancer achieved stable disease, partial response, or complete response [118]. These results may further underscore that antibody–drug conjugates (ADCs) targeting mesothelin are unlikely to revolutionize advanced pancreatic cancer therapy.

### Targeting angiogenesis-VEGF inhibitor plus chemotherapy regimens

Bevacizumab, a VEGF inhibitor, showed tolerability and clinical activity when combined with gemcitabine in patients with pancreatic cancer [119]. Additionally, heavily pretreated patients with advanced solid tumors demonstrated good tolerability and safety when treated with AG (albumin-bound paclitaxel and gemcitabine) plus bevacizumab. Among the 15 patients with pancreatic cancer, one achieved partial response with a 57% reduction in tumor size, and 10 (67%) had stable disease [120].

A phase I/II multicenter single-arm study of FABLOx (Metronomic 5-Fluorouracil Plus nab-Paclitaxel, Bevacizumab, Leucovorin, and Oxaliplatin) in patients with metastatic pancreatic cancer revealed the regimen's tolerability. The most common grade ≥ 3 adverse events were abdominal pain and fatigue. The ORR was 33%, with median PFS and OS of 5.6 (95% CI, 1.7–11.3) and 9.9 (95% CI, 4.4–13.2) months, respectively [121]. However, the phase II data alone are insufficient to draw positive conclusions, but the confirmation of safety in these trials has paved the way for subsequent clinical investigations.

In summary, monoclonal antibodies targeting cell growth signals have not shown superior efficacy in pancreatic cancer, even when combined with systemic chemotherapy. The potential of apoptosis-inducing strategies in combination with chemotherapy warrants further investigation, but the outlook may be uncertain at present. The effectiveness of targeting stroma and blood vessels in combination with chemotherapy remains unclear.

## Tumor vaccine

Tumor vaccines constitute a vital component of contemporary tumor immunotherapy, leveraging tumor cells or tumor antigen components to stimulate specific immune and humoral responses within the patient's immune system. This process enhances the body's ability to detect and combat tumors. Tumor vaccines encompass whole-cell vaccines, dendritic cell (DC) vaccines, peptide-based vaccines, and nucleic acid (DNA and mRNA) vaccines (Fig. 1). These vaccines differ in their mechanisms, and their combination therapies yield varying outcomes in clinical trials (Table 1).

### Whole-cell vaccines

Whole-cell vaccines involve the use of irradiated or otherwise treated self or allogeneic tumor cells, rendering them incapable of proliferation while retaining their immunogenicity. These vaccines, as a whole, can elicit anti-tumor immune responses within the body. Whole-cell vaccines offer multiple potential antigens, reducing the likelihood of antigen loss and enhancing immune responses to varying degrees.

#### Combination of whole-cell vaccine and chemotherapy: CRS-207 plus cyclophosphamide (Cy) plus GVAX

GVAX is a granulocyte–macrophage colony-stimulating factor (GM-CSF) gene-transfected tumor cell vaccine. It relies on two irradiated, GM-CSF-secreting allogeneic PDA cell lines. In the context of the GVAX pancreatic vaccine, low-dose cyclophosphamide was added as a neoadjuvant chemotherapy regimen, offering superior benefits over GVAX alone. Notably, the regimen induced tertiary lymphoid structures and anti-tumor immunological changes, such as Treg depletion, in most subsequent specimens. This suggests that vaccination can alter the "non-immunogenic" characteristics of pancreatic cancer by enhancing immune-infiltrating cells in the TME. However, the median survival of 4.3 months fell short of expectations [122, 123]. Nevertheless, given the observed immunological changes, the inclusion of CY when using GVAX appears to be a consensus.

Le DT and colleagues invested significant efforts in studying the combined therapeutic approach of GVAX and CRS-207, a tumor vaccine utilizing Listeria bacteria to activate the immune response to mesothelin. Their findings indicated that heterologous prime/boost with Cy/GVAX and CRS-207 as third-line therapy significantly improved OS in metastatic pancreatic cancer patients compared to Cy plus GVAX (6.1 months vs. 3.9 months; HR, 0.59; p = 0.02) in a phase II study [124]. Subsequently, after demonstrating the regimen's tolerability, the authors conducted a controlled experiment comparing this regimen to conventional chemotherapy. The results suggested that, in comparison with conventional chemotherapy, treatment with Cy/GVAX plus CRS-207 or CRS-207 monotherapy did not significantly improve survival. The data showed median OS values of 3.7 (95% CI 2.9–5.3), 5.4 (95% CI 4.2–6.4), and 4.6 (95% CI 4.2–5.7) months and PFS of approximately 2.2 months for all arms, including the group receiving chemotherapy [125].

Additionally, based on these two studies, the authors performed immunological analyses of patients with prolonged survival using single-cell mass cytometry and identified two cell subsets associated with improved OS: CD8( +)CD45RO(-)CCR7(-)CD57( +) cells and CD14( +)CD33( +)CD85j( +) cells. The former subset was more abundant, and the latter less so [126].

#### Combination of whole-cell vaccine and immune checkpoint drugs

The combination of tumor vaccines and ICIs has shown promise in preclinical models. This combination, along with low-dose cyclophosphamide and PD-1 blockade, can inhibit the immunosuppressive function of Tregs and the PD-1/L1 axis, enhancing the CD8 + T cell responses induced by tumor vaccines [127, 128].

However, the role of immune checkpoint antibodies in combination with whole-cell vaccines remains controversial. In another study by Tsuji Kawa T et al., Cy/GVAX and CRS-207 were combined with nivolumab. Although the experiment maintained a good safety profile, there was no significant improvement in OS, DCR, and median PFS in the experimental group compared to the control group without nivolumab (median OS: 5.9 [95% CI, 4.7–8.6] vs. 6.1 [95% CI, 3.5–7.0] months, HR: 0.86 [95% CI, 0.55–1.34]; DCR: 13.7% vs. 9.5%; median PFS: approximately 2.2 months for both arms). Moreover, the 12-month and 18-month OS rates trended less impressively in the experimental group compared to the control group [129].

In contrast, the combination of the pancreatic cancer GVAX vaccine (with low-dose cyclophosphamide), nivolumab, and urelumab (an anti-CD137 agonist antibody that enhances T-cell immunity) as adjuvant or neoadjuvant therapy in patients with resectable pancreatic cancer has demonstrated potential efficacy. In comparison with the group of patients receiving only the GVAX vaccine, those administered the three-drug combination exhibited a significant increase in disease-free survival (33.51 months vs. 13.90 months, HR = 0.55, *p* = 0.242) and overall survival (35.55 months vs. 23.59 months, HR = 0.59, *p* = 0.377). Additionally, an augmentation in activated cytotoxic T cells within the tumor was observed. However, this promising trial had certain limitations, including a smaller enrollment in the three-drug combination group and a higher proportion receiving adjuvant (m) FOLFIRINOX [130].

It is worth noting that patients treated with GVAX and the CTLA-4 antagonist ipilimumab experienced significant changes in the TCR repertoire [129]. In addition, ipilimumab treatment led to the expansion of clones associated with longer survival. However, when a phase II study of GVAX combined with ipilimumab was conducted, this regimen failed to improve OS compared to the addition of ICIs (HR: 1.85, 95% CI: 1.03–3.33, *p* = 0.036) and was prematurely closed. Nonetheless, improvements in immune cell infiltration following treatment were observed in both studies. Therefore, the combination therapy of tumor vaccination and ICIs warrants further investigation [131].

In conclusion, whole-cell vaccines, whether as monotherapy or in combination with immunotherapies, have not demonstrated superior therapeutic outcomes compared to traditional regimens. However, given the consistently low toxicity of immunotherapy and the advanced stage of disease progression in the primary study population, future developments in therapeutic strategies based on whole-cell vaccines hold promise.

### DC vaccine

DCs, known for their potent antigen-presenting abilities, serve as the crucial link between antigens and immune responses. DC-based anticancer vaccines employ native or patient-derived dendritic cells loaded with various forms of antigens in vitro to generate antigen-specific cytotoxic T cells. This approach results in the targeted destruction of tumor cells.

#### DC vaccine monotherapy

pMUC1-peptide pulsed dendritic cells (DCs):

Mucin 1 (MUC1), an extensively studied tumor-associated antigen (TAA), is aberrantly expressed in various malignancies [132–134]. In a phase I trial, autologous DCs pulsed with MUC-1 and subcutaneously administered to induce an immune response demonstrated safety in seven patients with advanced pancreatic cancer. Additionally, the vaccine induced anti-tumor immune responses against MUC-1[135].

Allogeneic lysate-dendritic cells (DCs):

In a phase I study involving a DC vaccine loaded with an allogeneic tumor cell lysate derived from multiple myeloma cell lines as adjuvant therapy, 10 pancreatic cancer patients who underwent surgical resection and received standard-of-care with no radiographic progression observed achieved an 80% 1-year disease-free survival rate. While the expected median OS and median PFS were not met, 70% of patients experienced no disease progression or recurrence during a median follow-up of 25 months [136].

#### DC vaccine plus chemotherapy plus immune cell therapy

Zoledronate-pulsed DCs (Zol-DCs) vaccine plus gemcitabine plus T cell therapy:

In addition to combination with chemotherapy, integrating other agents with distinct mechanisms enriches the therapeutic strategy for DC vaccines. Dendritic cells loaded with TAA were treated with zoledronate, a commonly used bisphosphonate in the clinic. This DC vaccine is known as the Zol-DCs vaccine and can promote the activation of Vγ9γδ T cells. A phase I/II study investigated a combination regimen of Zol-DCs with chemotherapy and intravenous infusion of αβT cells in 15 patients with locally advanced pancreatic cancer. Approximately 50% of patients achieved stable disease, with median PFS and median OS of 5.5 months and 12.0 months, respectively. Although these results were comparable to OS inpatients receiving standard gemcitabine-based chemoradiotherapy, the regimen demonstrated a 30.8% 2-year survival rate, outperforming patients who received chemoradiotherapy alone. Notably, improved survival correlated with a pre-treatment neutrophil/lymphocyte ratio (NLR) less than 5.0 and increased CD8 + /Treg ratio in post-treatment SD patients, suggesting their potential as biomarkers for Zol-DC immune combination therapy [137].

DC vaccine alone or DC vaccine plus lymphokine-activated killer [LAK] cell plus chemotherapy (gemcitabine with or without S-1):

In a retrospective study involving 49 patients with inoperable pancreatic carcinoma refractory to standard treatment, a combination therapy of DC vaccine alone or DC vaccine plus LAK cell and chemotherapy (gemcitabine + /S-1) resulted in 2 patients achieving complete response, five achieving partial response, and 10 achieving stable disease. The cohort exhibited a median survival of 360 days, suggesting potential for improving the prognosis of advanced pancreatic cancer patients. The addition of LAK cells enhanced treatment efficacy. Immunological analysis revealed that the reduction of regulatory T cells was closely associated with improved prognosis rather than an increase in tumor antigen-specific T cells [138]. These findings support the potential of combining cell therapy and chemotherapy with DC vaccines as a treatment option for pancreatic cancer patients.

#### DC vaccine plus adjuvant

Toll-like receptor (TLR)-3 agonist poly-ICLC plus DC vaccine (three distinct A2-restricted peptides):

Capitalizing on enhanced T cell responses observed at the cellular level when the TLR3 agonist poly-ICLC was combined with DCs, Mehrotra S et al. developed poly-ICLC as an adjuvant in combination with DC vaccine for 12 patients with metastatic (nine) or locally advanced unresectable (three) pancreatic cancer. Eight patients were assessed as having stable disease and progressive disease at day 56. The experimental median OS was 7.7 months; a notable improvement compared to the 4.2 to 4.9 months median OS achieved with second-line chemotherapy regimens for metastatic pancreatic cancer patients. This regimen was well-tolerated, with fatigue and self-limiting flu-like symptoms as the most common side effects. Although the experiment lacked data on immune changes in the tumor microenvironment post-treatment, the regimen exhibited superiority over existing standard treatment regimens [139].

#### Personalized neoantigen peptides (PEP-DC) plus nivolumab plus SOC chemotherapy plus aspirin

In a phase Ib trial, a novel proteo-genomic antigen discovery pipeline was designed for neoantigen prediction and selection. The trial combined nivolumab with chemotherapy in a treatment approach based on tumor vaccines. Aspirin was added to suppress immunosuppressive cells. CD4 + T cell responses against PEP candidates were detected in all three donors, although CD8 + T cell responses were not observed. This protocol highlights the feasibility of neoantigen vaccine production [140], offering innovative prospects for future vaccine treatment strategies.

While DC vaccine monotherapy has exhibited safety in earlier studies [135], its clinical benefits for pancreatic cancer have been modest. Conversely, combining DC vaccines with chemotherapy shows promise, and the inclusion of toll-like receptor (TLR)-3 agonists demonstrates favorable efficacy. The exploration of neoantigens in follow-up studies holds great potential for future vaccine treatment strategies.

### Peptide vaccines

Peptide vaccines, compared to other vaccine types, offer the advantage of lower toxicity and simplified synthesis. However, this ease of use comes with the potential for reduced immunogenicity. Hence, the active use of adjuvants or immunomodulators becomes crucial to elicit robust responses. Despite extensive clinical trials, peptide vaccines derived from sources such as telomerase have not demonstrated a significant clinical efficacy when compared to standard chemotherapy. Therefore, the potential of neoantigen vaccines holds promise for future developments.

#### Peptide vaccines monotherapy

Personalized peptide vaccination (PPV):

A retrospective study conducted in 2021 explored iNeo-Vac-P01, a personalized neoantigen-based peptide vaccine, in seven advanced pancreatic cancer patients with low tumor mutation burden (TMB). Concurrent administration of other treatments, including ICIs, was permitted. The vaccine was well-tolerated, with no significant vaccine-related adverse immune reactions reported. Patients exhibited a mean OS of 24.1 months, vaccine-related OS of 8.3 months, and PFS of 3.1 months. The DCR reached 85.71%, and the 1-year survival rate approached 50%. A substantial increase in antigen-specific TCR clones was observed in one patient who achieved long-term survival, suggesting iNeo-Vac-P01's potential to activate specific T cell subsets against tumor cells [141]. In summary, personalized peptide vaccines offer considerable clinical benefits, with immunological changes providing valuable insight for future research in this innovative strategy.

#### Peptide vaccines plus chemotherapy

Peptides WT1-pulsed dendritic cell vaccine combined with chemotherapy.

An early phase I study investigated the use of DCs pulsed with Wilms' tumor 1 (WT1)-specific peptides (DC/WT1-I, II, or I/II) in combination with gemcitabine for seven PDA patients. One patient achieved partial response, and the remaining six demonstrated stable disease. Notably, delayed-type hypersensitivity (DTH) responses specific to WT1 peptides were detected in four of the seven patients. Patients with a robust DTH response to WT1 peptide exhibited improved survival. The regimen also maintained a favorable safety profile, with grade 1 skin reactions at the vaccination site, mirroring those reported in previous gemcitabine trials. Additionally, vaccination promoted the long-term maintenance of WT1-specific memory CD8 + T cells [142, 143].

Yanagisawa R et al. conducted a phase I trial involving patients with surgically resected pancreatic cancer. The patient was administered a three-drug combination of antigen-pulsed DCs loaded with WT1 peptides (highly expressed in pancreatic cancer), S-1, and OK-432 (an adjuvant for the WT1-DC vaccine). No significant adverse effects were reported, and seven out of eight patients with surgically resected pancreatic cancer exhibited durable WT1-specific CTL immune responses. A 2-year OS rate of approximately 62.5% suggests its potential as a treatment option [144]. The regimen's superiority, stemming from these results, indicates its therapeutic potential, especially when combined with chemotherapy.

SVN-2B plus gemcitabine and/or tegafur/gimeracil/oteracil plus IFN beta(ADJUVANT).

A study involving 29 patients with metastatic pancreatic cancer, who had previously failed gemcitabine-based therapy, investigated KIF20A-66, an HLA-A24-restricted peptide vaccine derived from KIF20A. Among the patients who completed at least one course of treatment, 21 achieved stable disease, while eight experienced progressive disease [145]. This outcome underscores the potential activity of the vaccine.

Similarly, SVN-2B, another HLA-A24-restricted peptide vaccine with IFN-β as an adjuvant, was evaluated in 83 HLA-A24-positive pancreatic cancer patients. These patients were divided into three groups: SVN-2B plus IFN-β, SVN-2B alone, and a placebo group. Although differences in PFS and DCR were evident among the three groups, the variance in OS was less pronounced (102 days, 96.5 days, and 111 days, respectively; p = 0.4565). However, an increase in surviving 2B-specific CTLs was observed in the SVN‐2B plus IFN-β group, suggesting potential for long-term survival improvement [146].

OCV-C01 plus gemcitabine:

OCV-C01, comprising epitope peptides from KIF20A, VEGFR1, and VEGFR2, combined with gemcitabine resulted in a higher median DFS of 15.8 months in 30 resected pancreatic cancer patients (95% CI, 11.1–20.6), compared to gemcitabine alone (DFS 12.0 months). Furthermore, the DFS rate at 18 months reached 34.6% (95% CI, 18.3–51.6), with median OS not reached. The OS rate at 18 months was 69.0% (95% CI, 48.8–82.5). The combination was well-tolerated, with no significant differences in adverse effects between gemcitabine plus OCV-C01 and gemcitabine alone (*p* = 0.504). Nonetheless, this non-randomized trial and the low expression rate of KIF20A suggest the need for a larger sample size for more conclusive results [147].

Different combination regimens of peptide vaccines and chemotherapy exhibit varying efficacy among groups of pancreatic cancer patients. Overall, they demonstrate reliable safety profiles but fall short of significantly improving OS. These findings underscore the need to address performance limitations despite their favorable safety profiles**.**

### nucleic acid vaccine

#### DNA vaccine plus chemotherapy

Algenpantucel-L plus neoadjuvant SOC chemotherapy (FOLFIRINOX or gemcitabine/nab-paclitaxel):

Algenpantucel-L (AL) is an allogeneic pancreatic cancer vaccine designed based on the concept of hyperacute rejection. It consists of two human pancreatic ductal adenocarcinoma cell lines genetically engineered to express αGal through retroviral transfer using the murine αGT gene [148]. A recent phase III study aimed to assess the potential of AL as a neoadjuvant chemotherapy regimen to enhance patient outcomes. In this study, 303 patients with borderline resectable or locally advanced PDAC were divided into two groups. Group A received neoadjuvant standard-of-care chemotherapy (FOLFIRINOX or gemcitabine/nab-paclitaxel), while group B received the same standard neoadjuvant regimen with the addition of HAPa immunotherapy. The results indicated that there was no significant difference in median OS (14.9 months vs. 14.3 months; HR: 1.02, 95% CI 0.66–1.58; p = 0.98) and median PFS (13.4 months vs. 12.4 months; HR 1.33, 95% CI 0.72–1.78; p = 0.59) between the two groups [149]. It is evident that the incorporation of AL did not result in improvement.

#### DNA vaccine plus IL-12

Telomerase plays a pivotal role in tumor cell proliferation. The telomerase complex is essential for maintaining telomere length at chromosome ends during DNA replication. Human telomerase reverse transcriptase (hTERT), a catalyst within the telomerase complex, is highly expressed in tumors and promotes tumor growth by facilitating cell proliferation, epithelial-to-mesenchymal transition, and other pathways. Consequently, hTERT becomes a valuable anti-tumor target.

In a phase I trial evaluating the safety of DNA vaccines targeting hTERT in solid tumor patients, 34 pancreatic cancer patients who exhibited no evidence of disease (NED) after front-line therapy were enrolled in two groups. These groups received modified plasmid DNA encoding hTERT variants (INO-1400/INO-1401) alone or in combination with an interleukin 12 plasmid (INO-9012). Both groups displayed excellent tolerance. In terms of clinical activity, the median DFS was 9 months, with 41.4% of patients remaining disease-free at 18 months. Additionally, the production of hTERT-specific CD4 + and CD8 + T cells was observed in the remaining patients, which correlated with survival benefits [150].

#### RNA vaccine plus anti-PD-1 plus chemotherapy

RNA vaccines have garnered significant anticipation in recent years due to their relatively simple production process, high efficiency, and accuracy. A recent phase I clinical trial illuminated the potential application of neoantigen RNA vaccines in treating patients with surgically resected pancreatic cancer. In this clinical trial, Luis A. Rojas et al. sequentially administered atezolizumab, autologous neoantigen-specific RNA vaccine, and mFOLFIRINOX to patients, observing favorable tolerability with only one out of 16 patients experiencing grade 3 AEs. Additionally, the vaccine demonstrated the ability to induce high-intensity polyclonal neoantigen-specific T cell responses in 50% of patients, resulting in a longer median recurrence-free survival compared to non-responders (non-responders: 13.4 months vs. responders: not reached) at an 18-month median follow-up. This exciting outcome is evidently contingent on the presence of tumors [151]. The safety and effectiveness of this vaccine in pancreatic cancer patients await validation through larger-scale clinical trials. Nevertheless, this clinical trial underscores the feasibility of an mRNA-based neoantigen vaccine for treating pancreatic cancer [152].

Most of the vaccines evaluated in current clinical experiments have demonstrated good safety and tolerance. Relevant clinical data have shown certain immunological changes and clinical activity, resulting in varying degrees of disease remission. These effects are primarily reflected in the proportion of patients achieving stable disease and improvements in PFS. However, the impact on OS remains limited. To provide more reliable treatment options, it may be necessary to explore more diverse and precise directions.

## Adoptive cell transfer therapy

Adoptive cell therapy harnesses the body's own cells to enhance cellular resistance and eliminate rejection by re-engineering them. T cells, the cornerstone of immunotherapy, are extracted from the patient's body and genetically modified to express chimeric antigen receptors (CARs), enabling them to mount a more potent and sustained response. These engineered T cells circulate within the body, delivering precise and efficient anti-tumor capabilities [153] **(**Fig. 1**)**. While adoptive cell therapy has demonstrated remarkable clinical benefits in hematological malignancies [154], its effectiveness in solid tumors, with their complex tumor microenvironments, presents a challenge in predicting outcomes.

CD133, a transmembrane protein highly expressed in various solid tumors, including pancreatic cancer, emerges as a potential immunotherapy target for patients with advanced CD133-positive tumors [155]. Wang Y et al. investigated the efficacy of CD133-directed CAR-T cells in patients with advanced metastatic malignancies. Results from 23 patients, including 14 with hepatocellular carcinomas and seven with pancreatic carcinomas, showed that three achieved partial remission, and 14 achieved stable disease. The median PFS was 5 months, with the main observed toxicity being a decrease in hemoglobin/platelet counts [156]. Given the immunosuppressive nature of intrahepatic and pancreatic cancers, this regimen holds promise for pancreatic cancer patients.

Mesothelin, highly expressed in pancreatic cancer cells, represents a target for multiple immunotherapy approaches. In one phase I trial, engineered T cells were designed to transiently express a CAR specific for mesothelin. These cells were administered to six patients with chemotherapy-refractory metastatic PDAC. Of these patients, two achieved stable disease, with PFS ranging from 3.8 to 5.4 months. Monitoring of metabolic active volume (MAV) in individual tumor lesions revealed stability in three patients and a 69.2% decrease in MAV in one patient with confirmed mesothelin expression [157].

The authors also employed a lentiviral CAR-expression system to stably express CART-meso in T cells. This phase I trial included patients with malignant pleural mesothelioma (MPM), ovarian adenocarcinoma (OVCA), and PDAC. Results indicated that 11 of 15 patients achieved stable disease, with peak CART-meso cell numbers detected in peripheral blood at 6–14 days, although they persisted only transiently. Pre-treatment with cyclophosphamide enhanced CART-meso expansion but did not prolong persistence beyond 28 days. Additionally, CART-meso DNA was detected in 70% (seven of 10) tumor biopsies, and human anti-chimeric antibodies (HACA) were detected in the blood of 57.1% (eight of 14) patients. The limited clinical activity may be attributed to low mesothelin expression in the patient population. Overall, CART-meso cells demonstrated good tolerability and expansion in the blood of all patients but yielded limited clinical activity [158]. Furthermore, clinical trials evaluating mesothelin-specific CARs containing fully human scFv (NCT03054298 and NCT03323944) are ongoing to address concerns about murine scFv-induced immune clearance of CAR-T cells.

Human epidermal growth factor receptor 2 (HER2), a cell surface receptor highly expressed by tumor cells, has become a target for CAR-T cell therapy. In a phase I trial conducted by Feng KC et al., HER2-targeting CAR-T cells were administered to patients with advanced unresectable pancreatic cancer following pre-treatment with nab-paclitaxel and cyclophosphamide. The results demonstrated that two pancreatic cancer patients achieved stable disease, with PFS durations of 5.3 and 8.3 months, respectively. Notably, this data compare favorably to an overall median PFS of 4.8 months (range, 1.5–8.3 months) in the same group, suggesting that HER2-targeted CAR-T therapy may benefit specific pancreatic cancer patient populations [159].

Similarly, in another phase I trial [160], 16 patients with EGFR-positive metastatic pancreatic cancer underwent cyclophosphamide preconditioning followed by CART-EGFR cell therapy. Fourteen evaluable patients achieved partial responses lasting 2–4 months, and eight patients experienced stable disease. The median OS for all 14 evaluable patients was 4.9 months, with a median PFS of 3 months from the start of treatment. Notably, the occurrence of grade ≥ 3 adverse events, such as fever/fatigue, nausea/vomiting, and mucous membrane/skin toxicity, significantly improved after treatment. Overall, while the results were not ideal, they were encouraging.

Similarly encouraging efficacy was observed in seven locally advanced and metastatic pancreatic cancer patients with adoptive anti-CD3 x anti-EGFR bispecific antibody armed activated T cells (BATs). In this phase I/II clinical trial, seven patients with PC survived more than a year, including one who was still alive at 54 months. The median OS was 31 months, and two patients notably achieved CR after restarting chemotherapy [161]. In conclusion, BATs infusion is considered safe and may improve survival in patients with pancreatic cancer by inducing an adaptive anti-tumor immune response.

Despite the promise of adoptive cell transfer therapy, limited clinical trials exist due to the high cost and time-consuming nature of this approach (Table 1). Industrial production of off-the-shelf, universal CAR-T cells for patient treatment holds the potential to make this therapy more accessible. Additionally, improving the efficacy of this therapy in solid tumors will require further exploration. For pancreatic cancer, the challenges of antigen selection and the immunosuppressive microenvironment persist. However, monotherapy regimens continue to demonstrate clinical activity. Furthermore, cell therapies utilizing other immune effector cells such as NK cells and macrophages as carriers for CARs have shown promise in preclinical and clinical experiments [162, 163].

## Conclusion and discussion

It is evident that regardless of the chosen treatment modality, single-mechanism-based immunotherapy strategies face significant challenges in altering the grim prognosis of pancreatic cancer patients. Combining various immunotherapy approaches with conventional radiotherapy, chemotherapy, molecular targeted therapy, and other diverse treatment modalities has exhibited a more expansive realm of development and heightened therapeutic potential in clinical trials. This inclination is closely tied to the inherent attributes of PC itself.

The complex immunosuppressive microenvironment intrinsic to PC, compounded by the physical barriers erected by fibroproliferative stroma, and the distinctive immunological traits characterized by low TMB collectively exert a profound influence on pancreatic cancer's onset and progression. These factors impact multiple facets of the body's immune response against tumor cells. Strategies for future pancreatic cancer treatment must focus on mitigating or reversing these adversarial effects through comprehensive systemic treatment regimens.

Specifically, this involves deploying technical innovations such as constructing carrier systems to enhance drug delivery to tumor sites, breaching physical barriers, and conducting in-depth research into the complex pancreatic cancer tumor microenvironment to modify its immunosuppressive attributes. By doing so, we can unleash the full potential of anti-tumor immune cells like effector T cells, boosting their capacity to recognize and eliminate tumor cells.

Pancreatic cancer's low tumor mutational burden sets it apart from other solid tumors, resulting in fewer neoantigens, limited immune activation, and restricted options for targeting tumor cells. Additionally, tumor cell heterogeneity and the rarity of identical neoantigens across different individuals make it challenging to mount effective immunotherapies that target a single or a small number of neoantigens. These unique features and constraints make pancreatic cancer appear daunting to treat. However, a judicious combination of therapeutic strategies will undoubtedly enhance patient prognoses, paving the way for precise and personalized immunotherapy programs within the context of comprehensive treatment strategies.

The status and abundance of immune cells in the TME vary among different cancer types [164]. Considering the influence of the complex and unique tumor microenvironment of PDAC on immunotherapy and the diverse responses of different populations to tumor cells, further research on neoantigen vaccines is crucial to achieve personalized precision treatment strategies. Deepening our understanding of the correlation between different components in the TME of pancreatic cancer is essential. Adopting appropriate treatment plans tailored to patients with distinct TME characteristics or immune profiles is integral to personalized immunotherapy against pancreatic cancer.

Data from single-cell RNA sequencing revealed that, despite heterogeneity, immunosuppressive myeloid and macrophage populations predominate in the PDAC TME, with prevalent expression of immune checkpoints on dysfunctional T cells and NK cells [165]. Within the TME, the interactions of immune cells with other components such as the cellular matrix are complex. H. Sadozai et al. analyzed the composition of pancreatic cancer stroma and infiltrating immune cells, finding that immune cells may influence the PFS of patients with surgical resection of pancreatic cancer by altering the composition of pancreatic stroma. Levels of CD3 and CD206 can serve as prognostic indicators of PFS [166]. Another study also identified that long-term survivors (LTS) of PDAC exhibited higher infiltration levels of immune cells, particularly T cells, and positivity for TLS in the TME. Stromal iNOS cells and CD68 cells were considered to have greater prognostic value [167].

M. Wartenberg et al. integrated data on immune cell background and histological characteristics of patients with pancreatic cancer, identifying three subtypes with different immunological characteristics and prognosis: "immune escape," “immune enrichment,” and "immune exhausted." Approximately 35% of patients belong to the "immune enrichment” subtype, characterized by T cell enrichment, lower levels of FOXP3 + Tregs, and mutations in CDKN2A and PIK3CA, indicating a poorer prognosis [168].

Similarly, an analysis based on transcriptomic signatures of immune infiltration categorized PDAC into adaptive, innate, and immune-exclusion subtypes. Innate immune subtypes associated with poorer survival exhibited an enrichment of NK cells and neutrophils and an exclusion of other tumor-infiltrated lymphocytes. This suggests that therapies targeting NK cells and neutrophils may yield better therapeutic outcomes in these patients. The “T cell dominant” subtype, being the most immunogenic, demonstrated enrichment of adaptive immune-associated subsets, making it more suitable for immune checkpoint-related therapies. The “tumor dominant" subtype, characterized by low immune cell infiltration in the TME, indicates prominent metabolic adaptation [169].

The crosstalk between different components of the TME and immune cells is intricate. Identifying immunosensitive or drug-resistant patients through the analysis of tumor interstitial and immune backgrounds is crucial for predicting treatment responses [170]. Simultaneously, for patients who are not candidates for surgical removal, this approach is challenging but equally indispensable. In any case, promoting the infiltration of local anti-tumor immune cells and reversing the adverse effects of the tumor microenvironment on these cells are imperative to overcome the heterogeneous TME of pancreatic cancer.

Presently, several studies have identified immunosuppressive factors in pancreatic cancer that originate with gene mutations and intensify as tumors progress, ultimately culminating in the establishment of advanced local immune privilege. Preventing and detecting tumors early have always been paramount, particularly for diseases like pancreatic cancer that lack early symptoms and effective screening methods. Therefore, devising effective early screening strategies and corresponding interventions is of paramount importance. Furthermore, stratifying pancreatic cancer patients based on distinct genetic, pathological, and other attributes to tailor personalized treatments is another avenue worthy of exploration. Researchers have categorized patients into immune escape phenotypes, enrichment phenotypes, and exhaustion phenotypes based on molecular characteristics and prognosis, underscoring the inaccuracy of a one-size-fits-all treatment approach. Swift patient stratification via reliable early stage biomarkers and the selection of suitable individualized combination therapies may constitute a reliable future treatment option, albeit a challenging one.

In conjunction with several preclinical studies, we have observed consistent efficacy of oncolytic viruses against pancreatic cancer [171]. Additionally, targeting immune activation markers such as OX40 [172], CD40 [173], and 4-1BB [174], in combination with immunotherapy, consistently demonstrates potential therapeutic value. Moreover, an anticipated research direction involves enhancing the impact of immunotherapy by altering the pancreatic cancer TME with low immune invasion through matrix destruction or targeted interventions [175–177]. TLS, identified not only as a prognostic indicator of immunotherapy but also as inducible by immunotherapy under specific conditions, becomes an immune element in the anti-tumor immune response within tumors [178]. Given that the generation of TLS is invariably associated with the presence of high endothelial venules, the continual generation and maintenance of TLS at the tumor site can overcome the solid stromal barriers of pancreatic cancer, facilitating extensive immune cell infiltration and sustained anti-tumor immune responses.

Recent studies have illuminated the influence of the gut microbiome and its metabolites on the immune system in patients with pancreatic cancer [179]. Specifically, sachharopolyspora, pseudoxanthomonas, and streptomyces are significantly enriched in patients with long-term survival (LTS), promoting the recruitment and activation of T cells at the tumor site [180]. Beyond influencing the adaptive immune response, the gut microbiota can contribute to the progression of PDAC by regulating the innate immune system, particularly by inhibiting the infiltration and activation of NK cells [181]. A more profound understanding of the crosstalk between distinct intestinal environments and PDAC immune microenvironments may constitute a means to achieve personalized immunotherapy.

Simultaneously, the limitations of mouse models in pancreatic cancer research have become increasingly apparent. This underscores the imperative for more refined pancreatic cancer research models, emphasizing the disparities in experimental outcomes stemming from differences between human and mouse models. Exploring these distinctions holds the potential to deepen our comprehension of tumor immune mechanisms in pancreatic cancer.

Current clinical treatment and scientific investigation of pancreatic cancer face substantial hurdles. Overcoming these obstacles necessitates numerous endeavors and collaborations between researchers and clinicians. The joint dedication and cooperation of these stakeholders are indispensable in this endeavor.
